# Optimal Reopening Pathways With COVID-19 Vaccine Rollout and Emerging Variants of Concern

**DOI:** 10.3389/fpubh.2021.729141

**Published:** 2021-09-07

**Authors:** Yanyu Xiao, Shengyuan Chen, Yi Zhu, Zachary McCarthy, Nicola Luigi Bragazzi, Ali Asgary, Jianhong Wu

**Affiliations:** ^1^Department of Mathematical Sciences, University of Cincinnati, Cincinnati, OH, United States; ^2^Department of Mathematics and Statistics, York University, Toronto, ON, Canada; ^3^Laboratory for Industrial and Applied Mathematics, Department of Mathematics and Statistics, York University, Toronto, ON, Canada; ^4^Disaster and Emergency Management, School of Administrative Studies and Advanced Disaster and Emergency Rapid-Response Simulation, York University, Toronto, ON, Canada

**Keywords:** COVID-19, variants of concern, vaccine roll-out, optimal reopening pathway, optimization, mathematical model

## Abstract

We developed a stochastic optimization technology based on a COVID-19 transmission dynamics model to determine optimal pathways from lockdown toward reopening with different scales and speeds of mass vaccine rollout in order to maximize social economical activities while not overwhelming the health system capacity in general, hospitalization beds, and intensive care units in particular. We used the Province of Ontario, Canada as a case study to demonstrate the methodology and the optimal decision trees; but our method and algorithm are generic and can be adapted to other settings. Our model framework and optimization strategies take into account the likely range of social contacts during different phases of a gradual reopening process and consider the uncertainties of these contact rates due to variations of individual behaviors and compliance. The results show that, without a mass vaccination rollout, there would be multiple optimal pathways should this strategy be adopted right after the Province's lockdown and stay-at-home order; however, once reopening has started earlier than the timing determined in the optimal pathway, an optimal pathway with similar constraints no longer exists, and sub-optimal pathways with increased demand for intensive care units can be found, but the choice is limited and the pathway is narrow. We also simulated the situation when the reopening starts after the mass vaccination has been rolled out, and we concluded that optimal pathways toward near pre-pandemic activity level is feasible given an accelerated vaccination rollout plan, with the final activity level being determined by the vaccine coverage and the transmissibility of the dominating strain.

## Introduction and Background

COVID-19 has had tremendous impacts on public health and the economy globally. Non-pharmaceutical interventions (NPIs), including school and business closures, have been implemented in an effort to reduce transmission of COVID-19 and to protect those most vulnerable to severe disease (1). However, these NPIs do not come without associated social and economic losses. In the event of continually reducing case counts, hospitalizations, and mortality associated with COVID-19, decision-makers are posed with the task of identifying strategies to safely relax physical distancing measures. However, it is challenging to assess how and when a safe reopening can be executed, while minimizing public health and economic losses and at the same time ensuring the health care system will not be overwhelmed.

The circulation of more transmissible variants of concern (VOCs) has further made the identification of safe reopening strategies more complex. While efficacious vaccines exist and are being administered, most nations are in the situation where the wide rollout of vaccination among the general population has not been achieved; hence, most individuals remain susceptible to infection and controlling virus transmission (and hence the downstream hospitalizations and deaths) while more transmissible variants are actively circulating still poses a real challenge. In this light, the task of identifying smart reopening strategies provides an opportunity for mathematical modeling to assist and inform the reopening decision-making process through means of evidence synthesis.

In the Canadian province of Ontario, the first wave of COVID-19 occurred during the Winter and Spring months of 2020 and was controlled through the implementation of NPIs including so-called lockdown and stay-at-home measures (2–4). Previously, the optimal timings for Ontario's phased reopening that maximized the relaxation of physical contacts while not exceeding the province's ICU capacity for COVID-19 patients were identified through the integration of a disease transmission model with a stochastic optimization model (5). Following the premature easing of restrictions, Ontario experienced a resurgence of cases in Fall 2020 (“the second wave”) that triggered another lockdown which led to declining case counts, hospitalizations and associated mortality. This, along with a vaccine rolling out to at-risk groups and the surgency of a more transmissible variant under the surface of declining total cases, created the pressure to relaunch the reopening process as early as February 14, 2021. With the expectation of vaccines becoming available to a broader range of individuals in April 2021 and the potential start of mass vaccination for the general population, the identification of smart reopening strategies became critically needed at that time to minimize economic and social losses, and finding sub-optimal pathways has been remaining as a challenge when the optimal timing to relaunch the optimal reopening strategies was missed, and when mass vaccination rollout is being accelerated in parallel with the emergence of more transmissible variants.

Here we utilize the established optimization framework (5), updated according to the current scenario in Ontario, which includes the circulation of VOCs, the current Provincial vaccination program and its anticipated acceleration, as well as current estimated levels of contact mixing and case isolation interventions from the public health system to identify optimal staged reopening strategies that maximize the relaxation of physical contacts while considering the province's ICU capacity for COVID-19 patients. More specifically, we identify optimal strategies toward mass vaccination, had the optimal strategy been initiated on February 14, 2021, March 14, 2021, May 16, 2021 and June 14, 2021 that would enable a gradual increase in activity levels in the Ontario population. We examine the projected confirmed cases of COVID-19 and hospitalized cases including those in ICU according to each of the optimal reopening strategies; we then examine the situation when those opportunities were missed and then identify a new optimization opportunity facilitated by the accelerated vaccination rollout to allow for a pathway leading to near pre-pandemic activity level.

## Materials and Methods

### COVID-19 Pandemic in the Province of Ontario, Canada

The province of Ontario has implemented a variety of control measures with varying intensity in response to the circulation of SARS-CoV-2 virus in the population, as well as relaxations to partially resume social and economic activities. A series of physical distancing control measures were implemented in March 2020 to mitigate the first wave in the province. These restrictions began to be lifted in the province's staged reopening process from mid-May until October 10, 2020. At this time on October 10, 2020, control measures to mitigate the second wave were implemented in select hotspots in the province. On November 23, 2020, the enhancement of control measures had commenced, and this date marked the second so-called lockdown of Toronto and Peel. The modeling of these distinct phases of varying control measures (of first two waves) in terms of activity levels was conducted in prior transmission modeling works and the key event timelines discussed (2–4, 6). The first cases attributed to the B.1.1.7 lineage (Alpha), with demonstrated increased transmissibility, were detected in Ontario on December 26, 2020. Also, in late December 2020, there was a provincewide lockdown and also the schools went on Winter break. On January 12, 2021, a second state of emergency was declared and a stay-at-home order was effective as of January 14, 2021. In mid-February 2021, the majority of public health units in the province had lifted the stay-at-home order, while hotspots such as Toronto remained under these strict measures. These select hotspots had an active stay-at-home order which was effective until their eventual lifting in early March 2021 and then returned to the province's so-called response framework. In mid-March, daily confirmed cases began again to increase potentially due to increased social mixing and the circulation of more transmissible variant strains. In light of the reported case increase and circulating VOCs (namely B.1.1.7/Alpha lineage), a series of measures including a provincewide emergency brake was implemented in early April 2021. Days after followed a third state of emergency declaration and subsequent (second) stay-at-home order, in early April 2021. The stricter measures brought the “third wave” to a peak in mid-April 2021. Also, on April 23, 2021, the first cases of the B.1.617 lineage (Delta), substantially more transmissible than the B.1.1.7 (Alpha), were detected in the province. Finally, the stay-at-home order was lifted on June 2, 2021; however, restrictions remained in place to control transmission. The level of activities, the average level of contacts made by one individual per day, in these difference phases of the past three waves were estimated using a data fitting procedure described in the Supplementary Methods, and these estimated levels of activities, shown in Supplementary Figures 1, 2, provide important baselines for our model-based optimal reopening strategies.

### Transmission Dynamics Model

**Figure 1:**
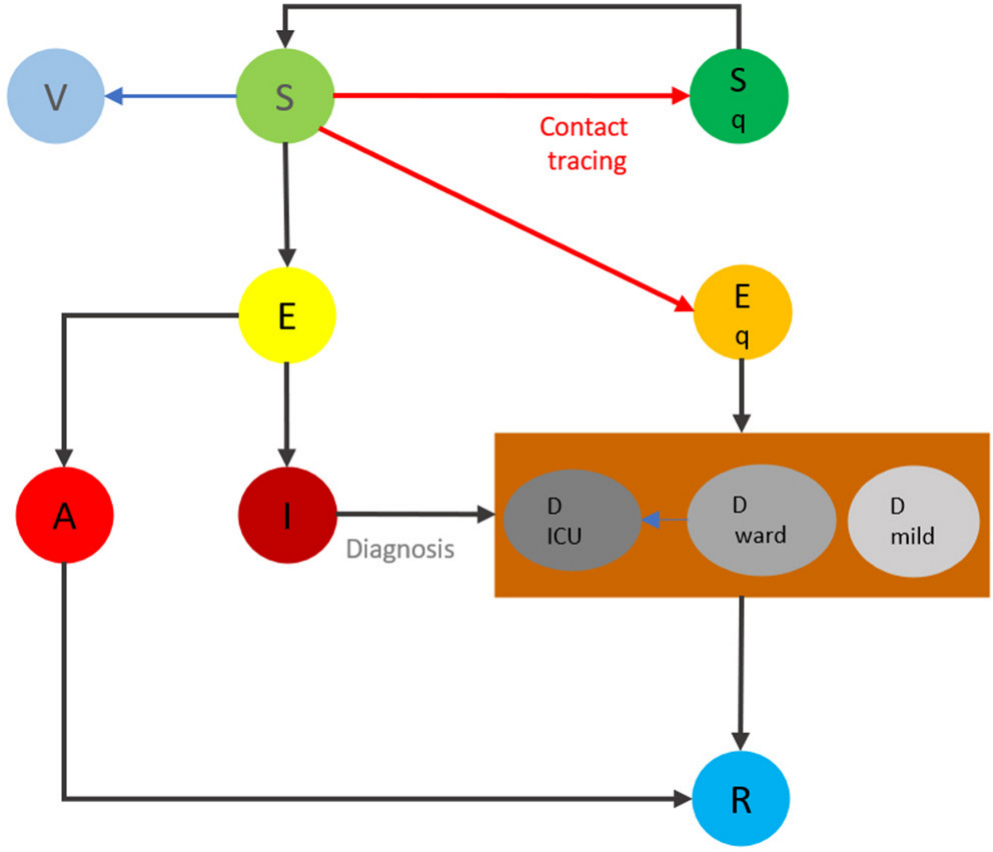
The flowchart of the transmission dynamics model, where the population is stratified into susceptible (*S*), vaccinated (V), exposed (*E*), asymptomatic infectious (*A*), symptomatic infectious (*I*), and recovered status (*R*), and by quarantine and isolation status (*S*_*q*_ and *E*_*q*_, respectively). The compartment of diagnosed but not yet resolved cases is further stratified by the need for the use of hospital wards (*D*_*ward*_) and/or ICU beds (*D*_*ICU*_), and mild (*D*_*mild*_).

We identified optimal strategies to increase the contact rate in the Ontario population toward a mass vaccination rollout, should the optimal strategy be initiated on February 14, 2021, March 14, 2021, May 16, 2021 and June 14, 2021. We accomplished this by using a stochastic optimization technology, based on a compartmental model of COVID-19 transmission dynamics [Supplementary Methods, Model Equations (1)] and key health care resources [Supplementary Methods, Model Equations (2)]. The diagram for the compartmental disease transmission model and health care resource model is shown in Figure 1. We obtained the baseline range of activity levels during different stages of reopening by fitting the model to the time series of the reported cases in Ontario from February 26, 2020 until April 11, 2021 and Supplementary Figure 1 shows the estimated average social contact rate *c* per day during this time period.

### Stochastic Optimization of De-escalation Plans

We investigated strategies to gradually relax physical distancing interventions, starting with (or without) school reopening, reopening of workplaces, and resumption of public events and activities. We set up the initiation time for reopening as February 14, 2021 and March 14, 2021 respectively to illustrate premature reopening impact on the existence and choice of optimal pathways. We searched for the optimal initiation time of physical distancing relaxation in different stages while keeping the number of COVID-19 patients needing ICU beds as small as possible. We first considered the situation in which the maximum allowable number of ICU beds in the province for COVID-19 patients was 350 and 500, respectively. We aimed to maximize the social contacts until the targeted mass vaccination rollout took effect in reducing the infection and disease burden, set here to be within May 2021. We aimed to find optimal strategies, in terms of the optimal time to switch between contact rates, by taking into account the various scenarios of social-economic activities at each reopening stage. We emphasize that in our study, the contact rates we defined in our transmission models are effective contact rates, similar as the contact rate modeled in (2). We assumed that the contact rates, at each stage are random variables, so the algorithm will inform the best possible contact rates at each stage and how long one should stay in a particular stage. The appropriate utilization of personal protective equipment (PPE) including face masks/coverings that would reduce effective contact rates is directly reflected in our data fitting for contact rates when the baseline transmission probability is fixed. We will discuss this in more details in the section **Discussion** as it relates to the optimal pathways identified.

For the optimization initiated on February 14, 2021 and March 14, 2021, we considered a de-escalation strategy to be acceptable if the projected number of ICU beds occupied by COVID-19 patients was within the health care system capacity during the period of cost-evaluation. We investigated strategies to gradually relax social distancing interventions, starting with school reopening, reopening of workplaces, and the resumption of public events and activities. The details of the model simulations and optimization setup for the February 14, 2021 initiation are included in Supplementary Methods. The contact rate ranges for each distinct phase of de-escalation and the parameters associated with transmission dynamics model parameters are included in the Supplementary Methods (Supplementary Tables 1–3). We note that in this simulation, the ICU beds available for COVID-19 patients was considered to be below 350.

In reality, however, Ontario started its reopening process on February 14, 2021, and relaxed measures earlier than the optimal strategy identified in the simulation above. In this light, updated stochastic optimization calculations showed that there was no longer a pathway that permitted the activity levels to be increased from the current level if the ICU beds available to COVID-19 patients were below 350. Therefore, we were forced to consider sub-optimal strategies with the ICU beds increased to 500 and a lower range of activity levels, as shown in Supplementary Table 4. In response, we ran optimization that accounted for the evolving situation and premature reopening to determine de-escalation strategies from the date of March 14, 2021. The details of the simulation are included in the Supplementary Methods.

To further demonstrate the application of our framework's ability to respond to the rapidly evolving pandemic situation in Ontario, we proceeded with additional simulations for optimal strategy identification. The first of which was initiated on May 16, 2021. In this set of scenarios, the vaccine rollout parameters were updated to reflect the vaccination program in Ontario (Supplementary Table 5). In the simulations, the contact rates for the de-escalations phases are shown in Supplementary Table 6. The contact rates in each de-escalation phase gradually increased until de-escalation phase 3 (the final phase), which has range [9.5, 10]. This final range was chosen, as these contact rates are representative of contact rates (or activity levels) close to the estimated pre-pandemic activity levels (4). The parameters associated with the diagnosed case compartments were also updated in this set of simulations (Supplementary Table 7). In Supplementary Table 7, the parameters associated with health care resources and model equations (2) are provided for both the resident and the VOC strains. Given that the ICU capacity for COVID-19 patients was exceeded in early April 2021, we no longer required strategies to meet the constraint that the projected COVID-19 cases in ICU remain below 500.

Finally, fourth and fifth optimization procedures were run, both of which initiating the reopening from a lockdown on June 14, 2021. The staged reopening process in this simulation was comprised of Phase 0: the activity level remains the same as that in late May for a few days; Phase 1: activity levels representative of rates estimated during Stage 2 reopening in Ontario; Phase 2: estimated Stage 3 reopening activity values; to finally Phase 3: estimated levels of activity in Ontario from the upper end of Stage 3 reopening to near pre-pandemic activity levels (4). The reference activity levels were chosen based on those estimated from a modified model based on the model shown in Figure 1 by considering two subgroups for vaccinated individuals, one for receiving vaccine within 14 days (and therefore still susceptible to infection) and the second subground is fully immunized, in the province [Supplementary Figure 2 and model equations (5)]. The contact rates for each stage are included in Supplementary Table 9. The fifth simulation had taken into account the emergence of the B.1.617 (Delta) lineage on June 1, 2021.

For the simulations initiating reopening on February 14, 2021 and March 14, 2021, the stochastic programming model (3) in Supplementary Methods was solved to minimize the intensity of reduced contacts. Similarly, for the simulations initiated on May 16, 2021 and June 14, 2021, the model (4) in Supplementary Methods was solved to minimize the intensity of reduced contacts.

## Results

### Pathways Should the Optimal Strategy Be Implemented on February 14, 2021

**Figure 2:**
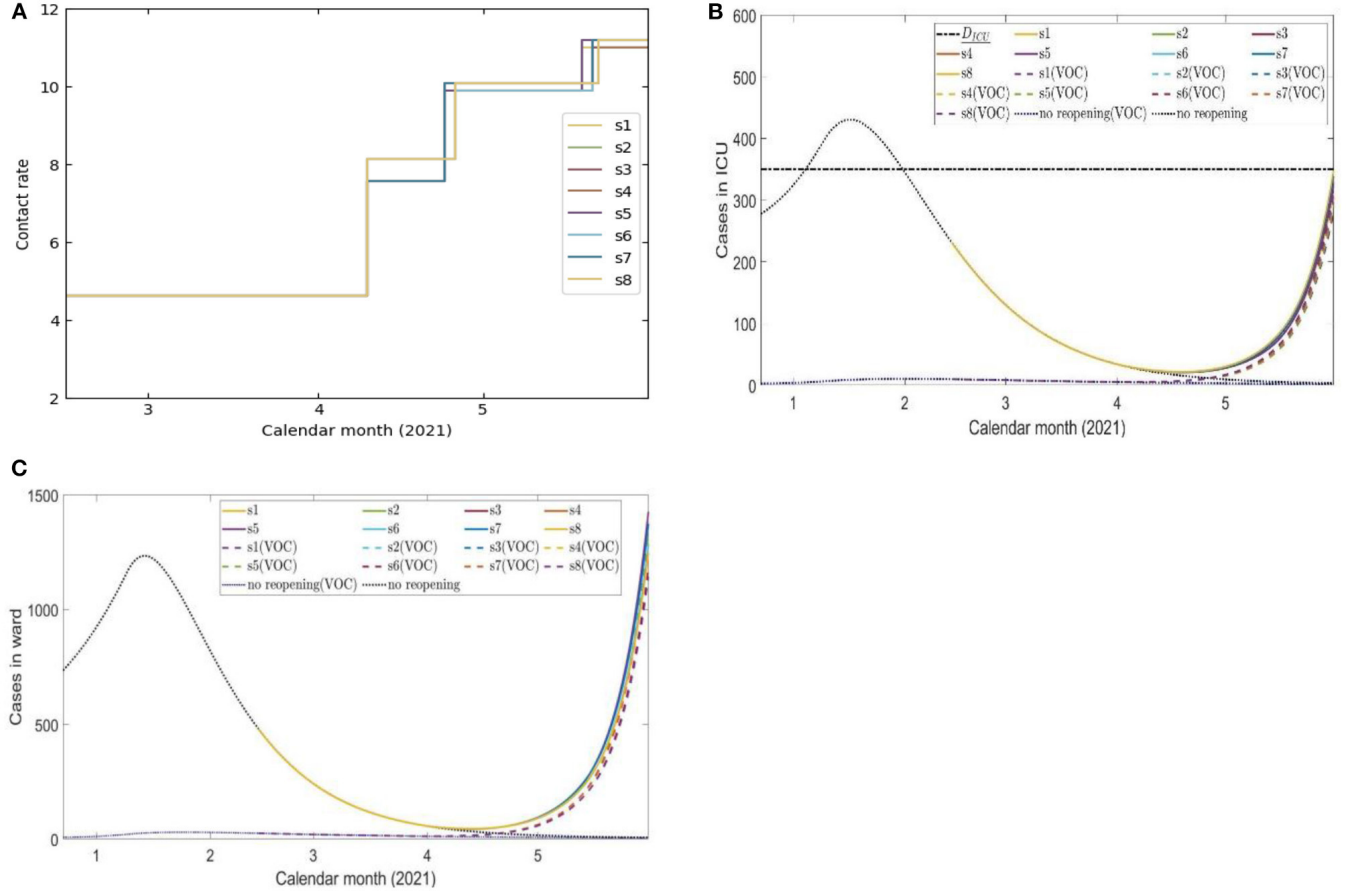
Eight scenarios of contact rates **(A)** and the projected number of COVID-19 cases in ICU beds **(B)** and ward beds **(C)** during four de-escalation phases of the optimal strategy initiated on February 14, 2021.

Figure 2 presents the optimal reopening strategy considering 8 different scenarios of the contact rates during the three reopening stages under the constraint of 350 ICU beds available for COVID-19 patients. The resulting duration of de-escalation phase 0 is approximately 56 days [ϵ_0_ = 55.28 (days)] (Supplementary Table 11). The duration of this phase is substantially longer than other phases identified in the optimization. This is because the number of ICU patients remains high in late January and early February 2021, as a result of the large number of infected individuals prior to phase 0 combined with the longer average ICU stay of patients. The optimal lengths of de-escalation phase 1 (ϵ_1_) are between 14 days and 16 days, the length of de-escalation phase 2 (ϵ_2_) varies from approximately 24 days to 27 days, depending on the scenario (Supplementary Table 11). Critically, the model-projected cases in ICU units remained to be less than the capacity by the time of May 31, 2021. Details of the optimal phase transition dates and scenario tree are shown in Supplementary Table 11 and Supplementary Figure 3, respectively.

### Pathways Toward Mass Vaccination Should the Optimal Strategy Be Initiated on March 14, 2021

**Figure 3:**
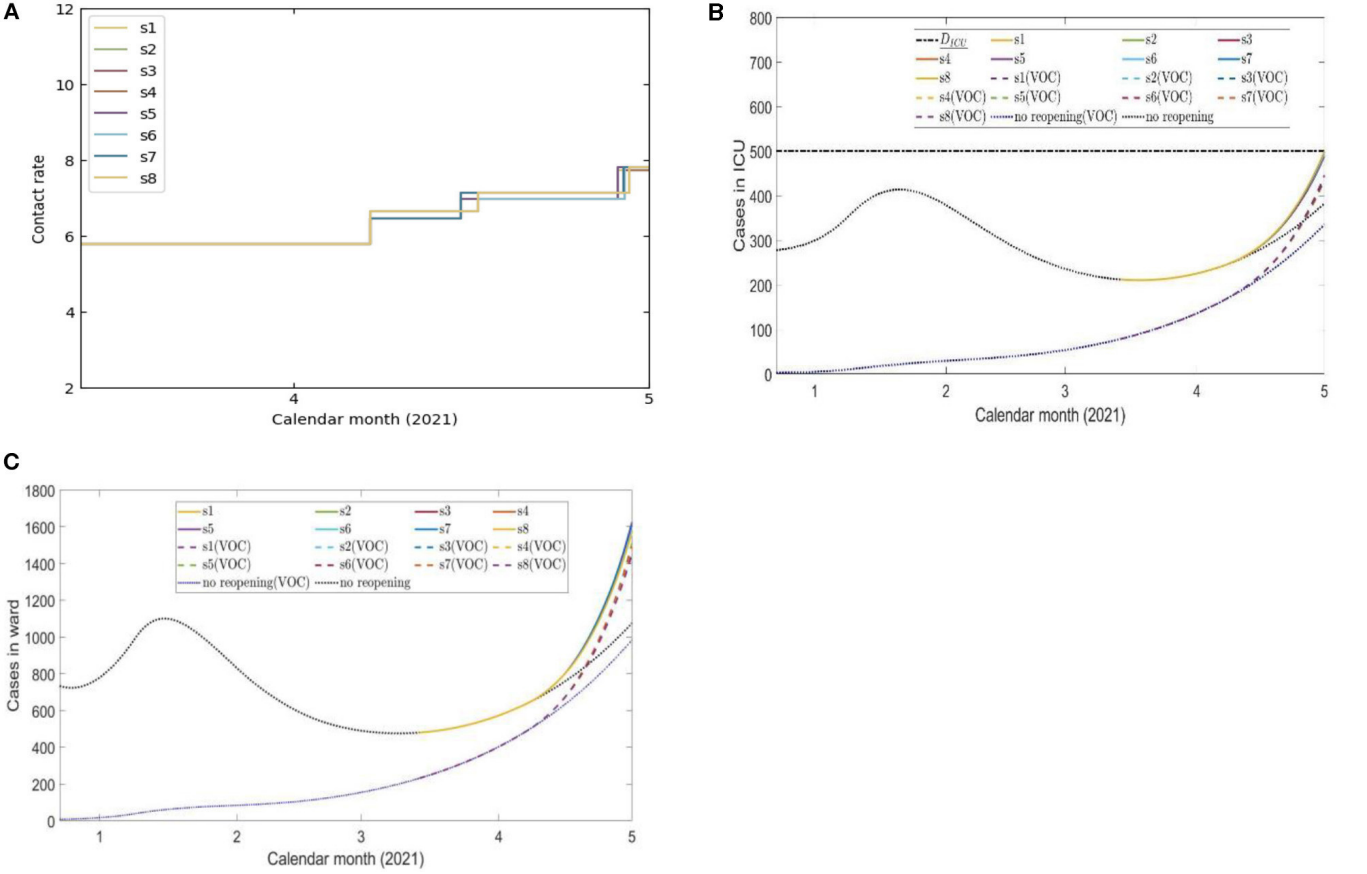
Eight scenarios of contact rates **(A)** and the projected number of COVID-19 cases in ICU beds **(B)** and ward beds **(C)** cases during 4 de-escalation phases of the optimal strategy initiated on March 14, 2021.

The optimal reopening strategies in terms of the contact rates, projected number of ward beds, ICU beds shown in Figures 3A–C. The simulation shows that there was a narrow pathway for the province to remain reopening and gradually increase the social-economic activities in the coming weeks until mass vaccination takes effect in the population. The simulation result shows that the province had to stay in stage 0 for approximately 25 days [ϵ_0_ = 24.47 (days)]. Details of the phase transition dates and the scenario tree are shown in Supplementary Table 12 and Supplementary Figure 5, respectively.

### Pathways Should the Optimal Strategy Be Implemented on May 16, 2021

The de-escalation phase duration, corresponding activity levels and date of initiation according to each scenario are presented in Supplementary Table 13. The scenario tree for these scenarios is shown in Supplementary Figure 7. All eight scenarios progress to phase 1 on May 24, 2021; the transition to phase 2 occurs on May 31, 2021, and the final transition to phase 3 occurred on June 7, 2021 (Supplementary Table 13 and Supplementary Figure 7) for each scenario. That is, the duration of de-escalation phase 0, 1, 2 were each 7 days (ϵ_0_, ϵ_1_, ϵ_2_ = 7) despite the modeled uncertainty in the contact rates.

**Figure 4:**
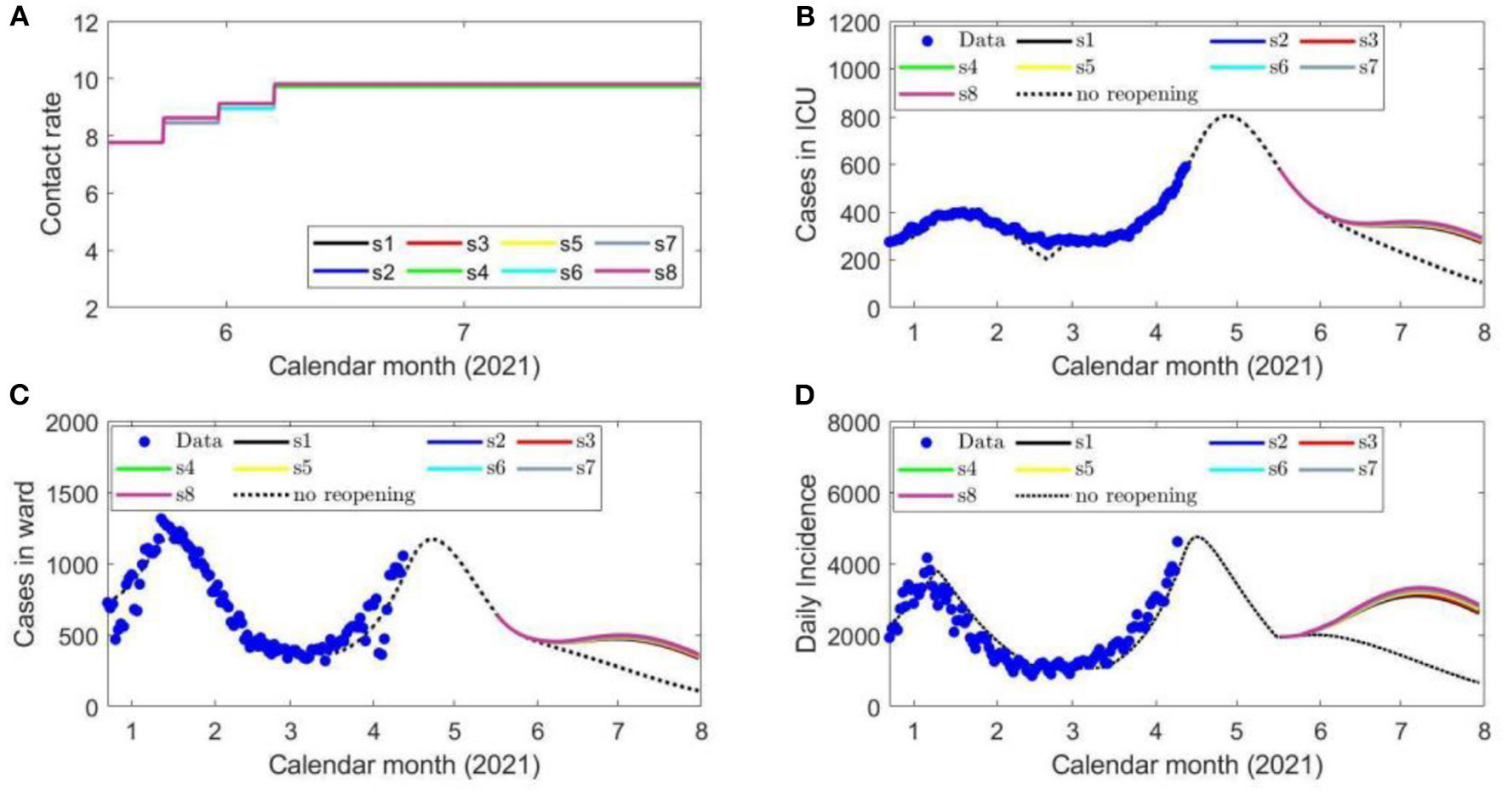
Eight scenarios of contact rates **(A)**, the projected number of COVID-19 cases in ICU beds **(B)**, ward beds **(C)** and the daily confirmed cases **(D)** during each de-escalation phase of the optimal strategy initiated on May 16, 2021. The blue circles represent the reported provincial data.

The contact rate according to each scenario is shown in Figure 4A. The projected number of COVID-19 cases until the end of cost-evaluation on July 31, 2021 when following the identified optimal strategies is shown in Figure 4D, as well as the projected COVID-19 cases occupying a ward bed (Figure 4C) and in the ICU (Figure 4B).

### Pathways Should the Optimal Strategy Be Implemented on June 14, 2021

**Figure 5:**
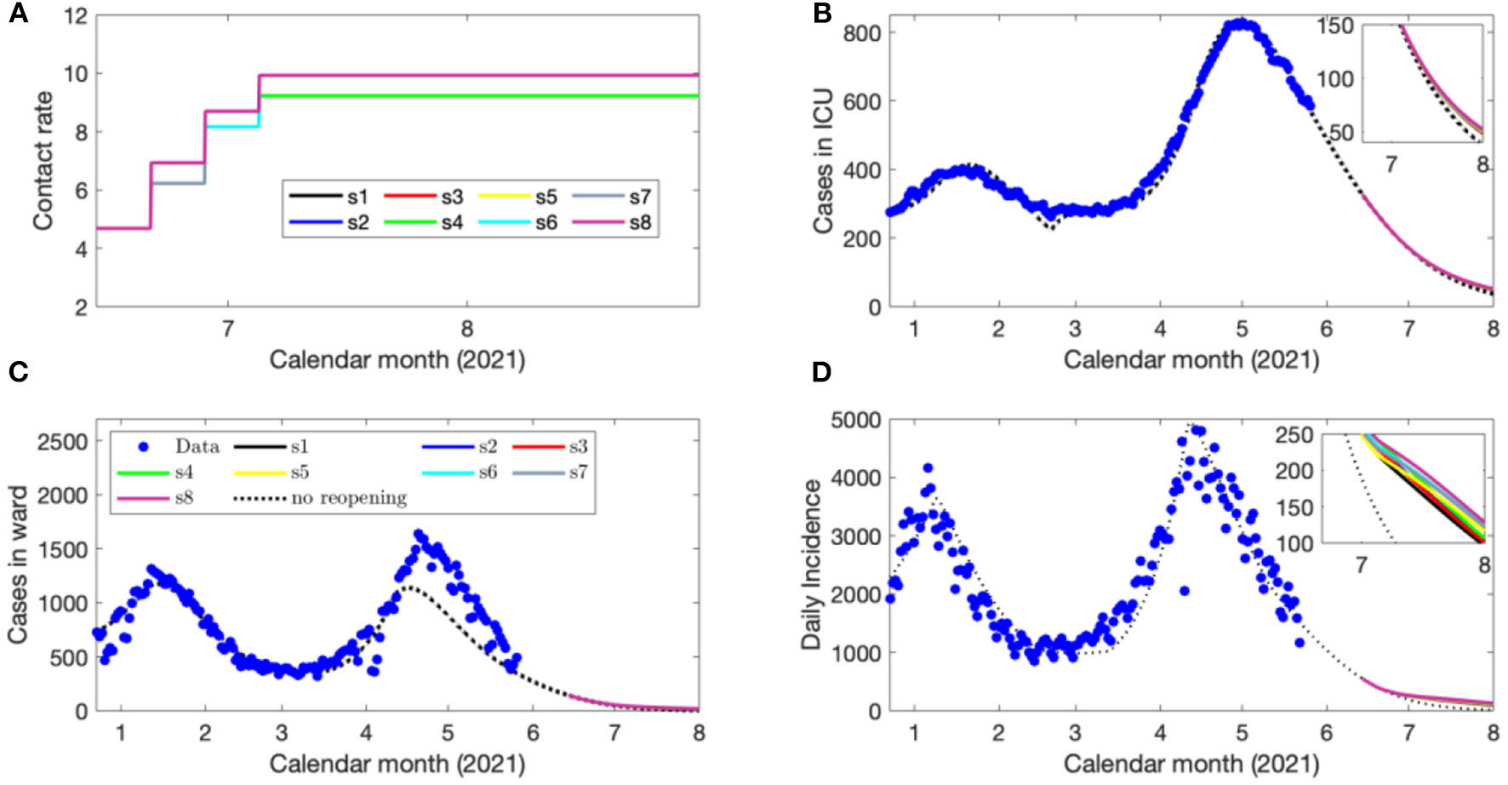
Eight scenarios of contact rates **(A)**, the projected number of COVID-19 cases in ICU beds **(B)**, ward beds **(C)** and the daily confirmed cases **(D)** during each de-escalation phase of the optimal strategy initiated on June 14, 2021.

The results from this simulation are shown in Figure 5 in terms of the contact rates (Figure 5A), projected number of ICU beds (Figure 5B), ward beds (Figure 5C) and daily confirmed cases (Figure 5D) when following the optimal strategy. All phase transitions were taken at the earliest time allowed of 7 days (Supplementary Table 14).

**Figure 6:**
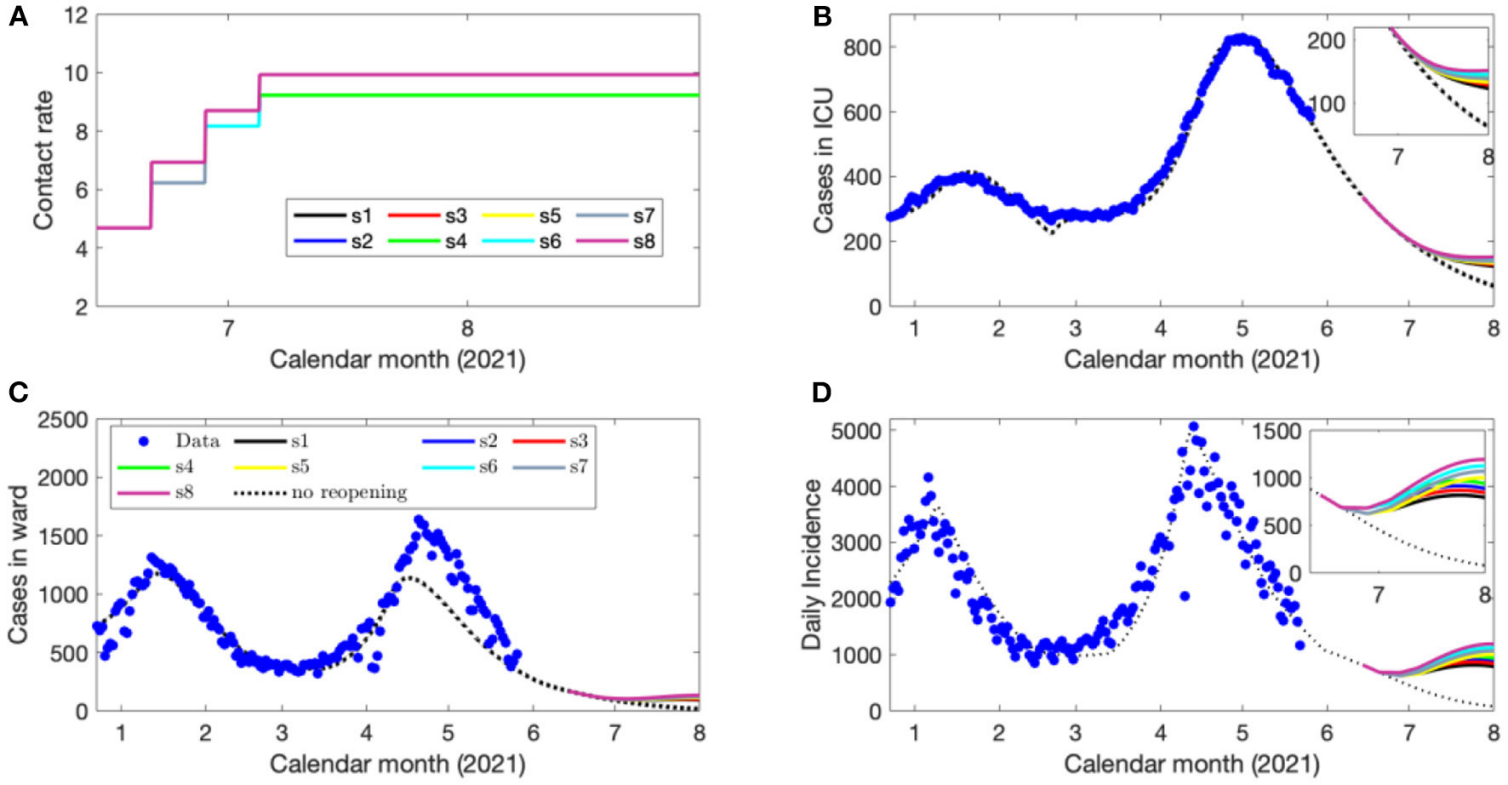
Eight scenarios of contact rates **(A)**, the projected number of COVID-19 cases in ICU beds **(B)**, ward beds **(C)** and the daily confirmed cases **(D)** during each de-escalation phase of the optimal strategy initiated on June 14, 2021 when B.1.617 emerged since June 1 2021.

We also considered the emergence of B.1.617 (Delta) in our optimal pathways since June 1, 2021, which has a higher estimated transmission rate shown in Supplementary Table 1. The results from this simulation are shown in Figure 6 in terms of the contact rates (Figure 6A), projected number of ICU beds (Figure 6B), ward beds (Figure 6C) and daily confirmed cases (Figure 6D) when following the optimal strategy. All phase transitions were taken at the earliest time allowed of 7 days (Supplementary Table 14).

## Discussion

We have utilized a disease transmission model and stochastic optimization model to identify reopening strategies in the province of Ontario, Canada from February 14, 2021 or March 14, 2021 that will minimize the loss of social contacts while the health system is not overwhelmed. Third, fourth and fifth sets of reopening strategies were identified from May 16, 2021 and from June 14, 2021 which considered the evolution of the outbreak situation and vaccination program in Ontario as well as the health system capacity. The third, fourth and fifth sets of simulations relaxed the constraint that the ICU beds for COVID-19 patients must be below 500 during cost-evaluation. In each simulation, we have considered a staged reopening approach composed of distinct de-escalation stages.

The first simulation shows that if the public health interventions such as contact tracing and case isolation remain at their estimated February 14, 2021-levels, and if this optimal strategy was adopted on February 14, 2021, the relaxation of physical distancing interventions could have been achieved without exceeding the ICU capacity until the vaccine became available to a broader range of individuals in Ontario (Phase 2 of Ontario's vaccination plan) (Figure 2). This required Ontario to stay in stage 0 for 56 days (Supplementary Methods, Table 11). The reopening strategies identified in this analysis accounted for the more transmissible B.1.1.7 (Alpha) lineage, which was (and is) actively circulating in the province, as well as the COVID-19 vaccinations occurring according to the most updated program.

Should this optimal strategy not be adopted at the beginning of lifting the lockdown, as was the case in Ontario, the choice of optimal pathways was much more limited but still feasible. The simulation based on the assumption that Ontario considered adopting this stochastic optimization technology on March 14, 2021 when the Province has advanced from lockdown for almost 1 month. Our simulation shows that there was no pathway toward mass vaccination taking effect in May 2021 if it is required that the activity level not reduced from its current level and if the ICU beds were limited to 350. We have run additional simulations to identify potential sub-optimal pathways when the COVID-19 patients needing ICU beds is increased to 500, and the maximal level of activities is below the estimated activity levels during Modified Stage 2 (Supplementary Figure 1). Our simulations do find sub-optimal pathways (Figure 3) and they are comparatively narrow.

As the ICU capacity for COVID-19 patients was exceeded in early April 2021, we ran a third set of simulations with strategy initiation on May 16, 2021 to reflect these new circumstances, also including the increased vaccine distribution and circulation of VOCs. We identified reopening strategies that enabled the contact rate from those estimated during lockdown to be increased to a final rate between 9.5 and 10 contacts per day per individual by June 7, 2021 (Supplementary Table 13). Critically, even at these relatively high levels of social contact representative of nearly the estimated pre-pandemic activity (4), the projected COVID-19 cases, hospitalizations, and cases occupying ICU beds declined in June 2021 as the vaccination program removes a sufficient number of susceptible individuals from the population (Figures 4B–D). The fourth simulation, initiating reopening on June 14, 2021 displayed similar features (Figures 5B–D), which demonstrates the impact of the vaccination program in the Province on COVID-19 transmission and its downstream effects to reduce the strain on the medical system. The fifth and final simulation, also initiating reopening on June 14, 2021, accounted for the circulation of the more transmissible B.1.617 (Delta) lineage. In this case, when following the identified optimal strategies, there was a projected spike in cases and hospitalizations which later subsides due to reduced number of individuals susceptible to infection (Figures 6B–D).

Despite modeled uncertainties in the contact rates for each of the de-escalation phases, the optimal timings identified in each scenario were similar for time of strategy initiation on February 14, March 14, May 16, and June 14, 2021 (Supplementary Tables 11–14). In the first set of simulations, the optimal initiation of the first stage was on April 10, 2021 for all eight scenarios (Supplementary Table 11). This first phase of de-escalation, lasting approximately 2 weeks until April 24–26, 2021 and the second phase of de-escalation lasting until May 19–22 2021, depending on the scenario (Supplementary Table 11). The third and final de-escalation phase was initiated between May 19–22, 2021 and marked activity levels near pre-pandemic levels (Supplementary Table 11). Note that in each simulated scenario, phase 0 has always a longer duration since the number of infected individuals prior to this phase were high and the time of ICU stay for ICU patient was relatively long. In these simulations, the ICU is predicted to reach its capacity for COVID-19 patients after May 31, 2021 (Figure 2B). Of note, the cases occupying the ward beds and ICU at the end of cost evaluation are composed primarily of VOC-associated cases (Figures 2B,C). Following the initiation of de-escalation phase 2 at the end of April, the increased contact rate results in the downstream effect of rapidly increased occupancy of ward beds and ICU during the month of May (Figures 2B,C). The projected rapid increase in ward bed and ICU occupancy at the end of cost-evaluation was also observed in the second set of simulations (initiation time on March 14, 2021), when following the optimal strategies (Figures 3A,C). This is in contrast to the simulations with initiation time of May 16 2021 and June 14 2021, where the impact of vaccination and reduction of individuals susceptible to infection was observed (Figures 4B–D, 5B–D) and the health care resources and confirmed COVID-19 cases were projected to decline. When the B.1.617 (Delta) was considered to have emerged since June 1 2021, with initiation time of June 14, 2021, the projected ICU occupancy was able to be controlled under 200, when following the optimal strategies (Figure 6B). There are several limitations of the analysis presented and areas that can be expanded in future work. The transmission model incorporated key interventions from the public health system, including the proportion of contacts of cases that traced and quarantined before they become infectious, and the rate at which symptomatic individuals are detected and isolated. However, in this work, we have assumed that these levels, as well as all transmission parameters remained at their current estimated levels until the end of cost evaluation in each simulation. In this light, during the period of cost-evaluation, the strain-specific transmission probabilities per contact remained at their respective levels estimated. Enhancing personal protection within the population (e.g., through the appropriate utilization of facial masks/coverings, other PPE and hygienic measures) can reduce the transmission probability per contact. High-efficacy face masks, for example, can reduce this transmissibility substantially (7). In our model, the parameter for the transmission probability achieved was estimated with a certain level of requirements for facial masks/coverings in public indoor settings in Ontario; however, a higher compliance/broader utilization with respect to masking could result in further reduced transmission probability and lead to a higher level of contact rate permitted. Instead of varying the transmission probability regarding to different level of personal behaviors during pandemics, we used the effective contact rates in our study that already considered the effort of personal behaviors (such as facial masks/covering utilization) in disease transmission. In a similar light, behavioral factors such as increased vaccination may lead to decreased risk perception and lower compliance levels or utilization with respect to PPE and hygienic measures, leading therefore to a higher transmission probability per contact. Meanwhile, relatively high reported case counts could lead to more cautious behavior including higher levels of PPE utilization and improved hygienic measures. These behavioral elements could also be incorporated in the model by considering the transmission probability per contact a dynamic function of vaccinations administered or reported case counts, similar to the established work (8). In this analysis, we extended the vaccination rate according to the expected rollout and did not explicitly consider hesitancy with respect to vaccination. It may be the case that, for some jurisdictions, additional considerations should be made to account for hesitancy (9). This could be modeled directly with anticipated uptake data [e.g., informed by surveys such as those reviewed in (9)] in the population or assumptions about the uptake. This may be particularly relevant for simulations over long time horizons. Also, the region of Ontario is composed of 34 public health units (PHUs) with varying infection rates and regional features, which we did not consider in this analysis. We here did not consider heterogeneities in the population such as age structure. We have considered the ICU capacity for COVID-19 patients not being exceeded as the sole constraint and the objective function being minimized represented contact loss. There may be additional factors to consider as constraints and within the objective function; however, in this analysis, we considered these two primary factors. In the model, for the vaccinated population we considered the “effectively vaccinated population” in the sense that effectively vaccinated individuals have 100% protection against infection. The daily vaccination rate therefore should be considered as the “effective daily vaccination rate”: the product of vaccine doses administered times the vaccine efficacy against infection. This consideration can be replaced by more explicitly incorporating “leaky” or “all-or-nothing” vaccines, for example, in future studies (10). Also, in reality, there are several different vaccine products approved for use (and being administered) in Ontario and therefore there are variations in vaccine efficacy according to product. Similarly, for select vaccination regimen, individuals receive a first dose followed by a second dose at a later date. These two elements can be explicitly incorporated in future studies. Finally, in light of limited data regarding the duration of protection granted by vaccination, we have assumed no waning effect of the protection granted.

The optimization framework established in our prior work (5) and extended here is flexible and amenable to further expansion. Potential future directions include the incorporation of key heterogeneities in the population such as age structure. The stochastic optimization framework can accept an age-structured analog of the transmission model used in this analysis, which features age- and setting (household, workplace, community and school)- specific contact mixing (4). Thus, optimal pathways with additional considerations for age-specific activity levels could be identified. In a similar light, age-structured transmission models are equipped to weigh different vaccination strategies; for instance, according to their capacity to interrupt transmission and reduce mortality (11). The stochastic optimization technology demonstrated within can be applied also to weigh these vaccination rollout strategies (e.g., including age prioritizations) according to the program's priority needs. Prior work has made the case for the strong coupling of social and epidemiological dynamics (11). As an example, the perception of risk may be linked to the population's collective adherence to NPIs (11). Hence the consideration for social factors by incorporating social dynamics (coupled with epidemiological dynamics) may provide additional insights with respect to reopening strategies. The modeling and quantification of abstract behavioral factors such as the population's level of caution and sense of safety may also be considered (8). We here have identified reopening strategies according to activity levels based on the anticipated rollout to the population. In addition to the identification of reopening strategies, we note that an effective implementation requires additional considerations given the critical role of the population's collective behavior on the disease trajectory. In this light, understanding how to effectively reach, communicate with and build trust with the public is key. Overall, the incorporation of social dynamics may uncover additional insights to equip decision-makers with, in order to inform suitable actions from the public health system to mitigate social and economic burden.

We have expanded our previously established study to incorporate the circulation of the VOCs circulating in Ontario, and parameterized the transmission model according to the current circumstances (including ongoing vaccination efforts and circulation of VOCs), in order to identify optimal pathways out of the restrictions starting on February 14, 2021, March 14, 2021, May 16, 2021 and June 14, 2021 while considering the health system capacity. We note the generality of this method; the transmission model can be parameterized according to different regions and the contact levels for reopening phases may also be altered based on regional constraints. In this sense, we have developed and illustrated a methodology with the intention that it can be utilized to identify reopening measures in different regions. We further have demonstrated the practical usage of this framework through this retrospective analysis, which allowed for the identification of the updated optimal pathways according to evolving circumstances.

## Data Availability Statement

The original contributions presented in the study are included in the article/Supplementary Material, further inquiries can be directed to the corresponding author/s.

## Author Contributions

YX, SC, AA, and JW: conceived the experiment. YX, SC, YZ, ZM, AA, and JW: run the experiment and analyzed the data. YX, SC, YZ, ZM, NB, AA, and JW: drafted and revised the manuscript. All authors contributed to the article and approved the submitted version.

## Funding

This project has been partially supported by the Canadian Institute of Health Research (CIHR) 2019 Novel Coronavirus (COVID-19) rapid research program.

## Conflict of Interest

The authors declare that the research was conducted in the absence of any commercial or financial relationships that could be construed as a potential conflict of interest.

## Publisher's Note

All claims expressed in this article are solely those of the authors and do not necessarily represent those of their affiliated organizations, or those of the publisher, the editors and the reviewers. Any product that may be evaluated in this article, or claim that may be made by its manufacturer, is not guaranteed or endorsed by the publisher.
