# Supplementary material for: Optimal Reopening Pathways With COVID-19 Vaccine Rollout and Emerging Variants of Concern
Source: Front Public Health. 2021 Sep 7;9:729141. doi: 10.3389/fpubh.2021.729141 (PMC8452896; doi:10.3389/fpubh.2021.729141)
Supplement: Supplementary file 1 [file Table_1.pdf]

1 Supplementary Methods

1.1 The Transmission Dynamics Model

We developed a stochastic approach [5], based on a compartmental model describing COVID-19 transmission dynamics. This model incorporates public health measures that have been used globally, in addition to the usual stratification of the population by their infection status, including both asymptomatic and symptomatic infections. The public health measures include detection of cases by testing and their isolation, tracing of contacts with cases and their quarantine and isolation. In the model, the population is divided into susceptible (S), exposed (E), asymptomatically infectious (A), infectious with symptoms (I), and recovered (R) compartments according to the clinical and epidemiological status of individuals. We also include diagnosed and isolated (D), self-isolated susceptible (S_q), and isolated exposed (E_q) compartments based on control interventions. Within the modelling framework, we use a parameter q to reflect the effectiveness of contact tracing and quarantine, this parameter is the proportion of individuals exposed to the virus who are traced and (effectively) quarantined (referred to as the quarantine proportion, and effectiveness means these individuals are removed from the transmission chain). Quarantined individuals can either move to the compartment E_q or S_q, depending on whether transmission has occurred (with probability q), while the other proportion, $1-q$, consists of individuals exposed to the virus who are missed from contact tracing and, therefore, move to the exposed compartment E if infected, or stay in the compartment S otherwise. The transmission dynamics model is

$$\begin{aligned}
S' &= -\big(\beta c + cq(1-\beta)\big)\frac{S(I+\theta A)}{N} + \lambda S_q - \chi,\\
E' &= \frac{\beta c(1-q)S(I+\theta A)}{N} - \sigma E,\\
I' &= \sigma\rho E - (\delta_I + \alpha + \gamma_I)I,\\
A' &= \sigma(1-\rho)E - \gamma_A A,\\
S_{q}' &= \frac{(1-\beta)cqS(I+\theta A)}{N} - \lambda S_q,\\
E_{q}' &= \frac{\beta cqS(I+\theta A)}{N} - \delta_q E_q,\\
D' &= \delta_I I + \delta_q E_q - (\alpha + \gamma_D)D,\\
R' &= \gamma_I I + \gamma_A A + \gamma_D D,
\end{aligned} \tag{1}$$

where N denotes the total population.

The aforementioned model has been developed in a series of studies, parametrized by fitting to the incidence data in the Province of Ontario, Canada [2, 3], and has been successful in predicting (two weeks ahead) the peak timing (around April 16th, 2020) and the peak value of COVID-19 cases for the first wave in the Province of Ontario. This projection was adopted by the Ontario government at the time, and the estimation of the necessary public health interventions, including testing speed, quarantine and isolation proportions for Ontario and Canada to relax certain social distancing measures for

reopening post the first wave was used by both the Ontario provincial and Canadian federal public health agencies. The model-based and stochastic optimization technique-supported recommendation for optimal reopening pathways (timing and mode for different stages) was not adopted: based on our simulation and analysis published in [5], the optimal timing for Ontario to advance to Stage 2 reopening would be late September 2020, although the Province advanced to Stage 2 much earlier than this optimal strategy recommended. This aggressive and premature increase in the activity level led to the third wave in Ontario.

To have a baseline range of activity levels during different stages of reopening, we first fitted the model to the time series of the reported cases in Ontario from February 26, 2020 until April 11, 2021. Supplementary Figure 1 below presents the estimated average social contact rate c per day.

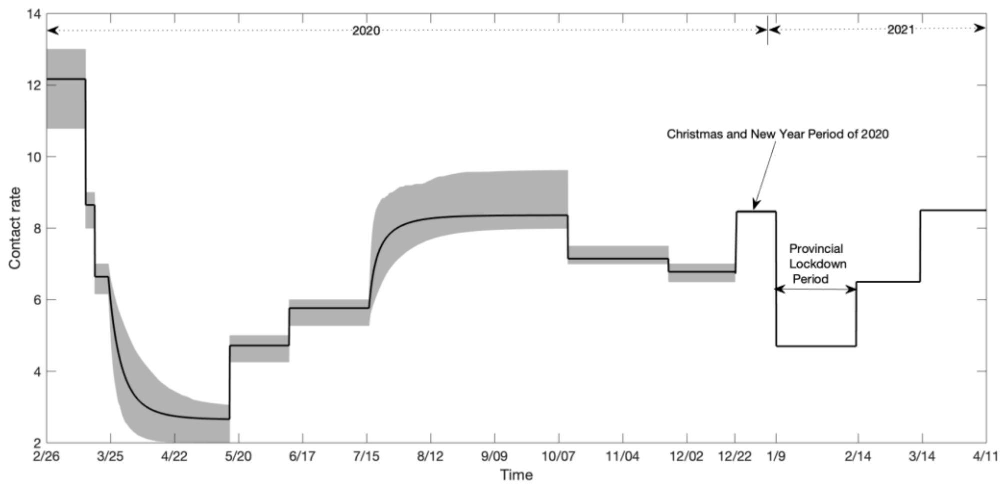

Supplementary Figure 1. Estimated activity levels in Ontario during different phases of physical distancing measures (February 22, 2022-April 11, 2021), using the baseline transmission dynamics model.

As experienced in the second wave, the availability of key medical resources including hospital wards and ICU beds plays an important role in determining whether social distance enhancement including lockdown to revert to early stages is needed. Therefore, we further divide compartment $D(t)$ (those diagnosed but not-yet-recovered individuals) as follows:

$$D = D_{mild} + D_{ICU} + D_{ward},$$

where D_{mild} is the compartment of confirmed cases who show only mild symptoms and do not require hospitalization, D_{ICU} is the compartment of those who are hospitalized in the ICU with severe symptoms, and D_{ward} is the compartment of those who are hospitalized in non-ICU units. The disease progression involving the medical treatment process is described as follows:

$$\begin{aligned} D'_{mild} &= (1-h)\big(\delta_I I + \delta_q E_q\big) - (\alpha + \gamma_{mild})D_{mild}, \\ D'_{ICU} &= h(1-w)\big(\delta_I I + qE_q\big) + b_{ward}D_{ward} - (\alpha + \gamma_{ICU})D_{ICU}, \qquad (2) \\ D_{ward}{}' &= hw(\delta_I I + \delta_q E_q) - (\alpha + \gamma_{ward} + b_{ward})D_{ward}. \end{aligned}$$

Here, h is the proportion of hospitalized cases among newly confirmed cases, among which a proportion w are hospitalized in non-ICU units directly. Using data fitting, we obtained that $h = 0.0397$ and $w = 0.9513$ before February 14, 2021; and $h = 0.0342$ and $w = 0.8731$ after February 14 and till April 12, 2021. The recovery rates for individuals in each state are denoted by mild, ICU and ward. Here, we use values from [12, 13, 14], namely, $\gamma_{mild} = 1/5$ and $\gamma_{ward} = 1/12$. For the recovery rate of individuals in ICU, we have distinguished the differences for wild and VOC strains, and used the fitted results γ_{ICU}^{w} =0.1431, γ_{ICU}^{VOC} =0.0876 and γ_{ICU}^{w} =0.0933, γ_{ICU}^{VOC} =0.0853 before and after February 14, 2021, respectively.

Supplementary Table 1: Parameters for COVID-19 transmission dynamics in Ontario, Canada. Note: *: estimated from data fitting of ICU and ward between December 23, 2020, to February 14, 2021. **: calculated from the average of Ontario vaccines administered daily data.

Parameter	Definition	Value
c	Contact rate between December 23, 2020 to January 9, 2021	8.4644
q	Fraction of quarantined exposed individuals before February 14, 2021	0.3949
σ	Transition rate of exposed individuals to the infected class	1/5
λ	Rate at which the quarantined uninfected contacts were released into the wider community	1/14
ρ	Probability of having symptoms among infected individuals	0.7036
δ_I	Transition rate of symptomatic infected individuals to the quarantined infected class	0.1378
δ_q	Transition rate of quarantined exposed individuals to the quarantined infected class	0.1237
γ_I	Recovery rate of symptomatic infected individuals	0.1627
γ_A	Recovery rate of asymptomatic infected individuals	0.139
γ_{mild}	Recovery rate of diagnosed individuals with mild symptoms	0.2
γ_{ICU}	Recovery rate of individuals in ICU units	1/14
γ_{ward}	Recovery rate of individuals in non-ICU units	1/12
α	Disease-induced death rate	0.008
θ	Modification factor of asymptomatic infectiousness	0.0275
h^*	Proportion of hospitalized cases among newly confirmed cases	0.0397
w^*	Proportion of cases admitted to non-ICU units among newly hospitalized cases	0.9513
$b_{ward}{}^*$	Rate of transfer from non-ICU units to ICU units	0.0245
β^{NV}	Probability of transmission per contact (non-VOC)	0.1073
β^{V}	Probability of transmission per contact (B.1.1.7 Strain)	0.1502
β^{B1617}	Probability of transmission per contact (B.1.617 Strain)	0.2103
χ^{**}	Effective of Vaccines administered per day from December 23, 2020 to February 14, 2021	4831.38
χ_r^{**}	Effective of Vaccines administered per day after February 14, 2021	10070.91

l	The rate of vaccinated individuals acquiring immunity	1/14

Supplementary Table 2: Initial conditions for the baseline COVID-19 transmission dynamics model with medical resources.

Initial values (non-VOC)	Definition	Value
$S(0)$	Initial susceptible population	1.4203344×10^7
$E(0)$	Initial exposed population	1.1774×10^4
$I(0)$	Initial symptomatic infected population	5.4303×10^3
$A(0)$	Initial asymptomatic infected population	4.4979×10^3
$S_q(0)$	Initial quarantined susceptible population	1.6175×10^5
$E_q(0)$	Initial quarantined exposed population	1.2332×10^4
$D_{mild}(0)$	Initial diagnosed population with mild symptom	1.8422×10^4
$D_{ICU}(0)$	Initial diagnosed population in ICU units	2.75×10^2
$D_{ward}(0)$	Initial diagnosed population in non-ICU units	7.27×10^2
$R(0)$	Initial recovered population	2.9052×10^5

1.2 Stochastic Optimization of De-escalation Plans

We investigate a strategy to gradually relax social distancing interventions, starting with school reopening, reopening of workplaces, and resumption of public events and activities. We set up the initiation time for reopening as February 14th and March 14th, 2021 respectively to illustrate premature reopening impact the existence and choice optimal pathways.

We look for the optimal initiation time of social distancing relaxation in different stages while keeping the number of COVID-19 patients needing ICU beds below a certain threshold. In our study, we consider the situation that the number of ICU beds in the province can be 350 and 500. We aim to maximize the contacts until the targeted mass vaccination rollout takes effect in reducing the infection and disease burden, set here within to be May 2021.

In what follows, we describe the setup for the optimal strategy identification for the February 14th, 2021 initiation time. The subsequent simulations (March 14th, May 16th and June 14th) and their slight variations on this setup are described afterward. We aim to find an optimal strategy, in terms of the optimal time to switch between contact rates, by taking into account the various scenarios of social-economic activities at each reopening stage. We emphasize that in our study, we assume that the contact rates at each stage are random variables, so the algorithm will inform the best possible contact rates at each stage and how long one should stay in a particular stage.

We consider four time-points, t_0, t_1, t_2 and t_3, each of which corresponds to the beginning of a particular stage. Each reopening stage is characterized by a contact rate, with $c_{r,0}$, $c_{r,1}$, $c_{r,2}$ and $c_{r,3}$ denoting the contact rates in each reopening stage (0, 1, 2 and 3). Symmetric triangular distributions are defined on the ranges presented in Supplementary Table 3.

In the first simulation, we set up the maximum level of contact rate to be the one at the pre-pandemic level and assume that $\underline{c_{r,3}} = 11.58$. The ranges of each triangular distribution are chosen from [2]. They are calculated by modifying weights of four different contact matrices at households, workplaces, schools, and communities-and others according to strategies of relaxing social distance in different reopen stages in Ontario, Canada.

Supplementary Table 3: Parameters for COVID-19 transmission dynamics, post reopening after the second wave in Ontario, Canada. VOC is considered, with the initial value of VOC being 1% of the corresponding values for $E, I, A,\ S_q,\ E_q, D_{mild}, D_{ICU}, D_{ward}$.

Parameter	Definition	Value
c	Contact rate between January 9 and February 14, 2021	4.6
$c_{r,0}$	Contact rate during reopening stage 0 (since February 14, 2021)	4.6
$[\underline{c_{r,1}}, \underline{c_{r,1}}]$	Range of the contact rate during reopening stage 1	[6, 9.394]
$[\underline{c_{r,2}}, \underline{c_{r,2}}]$	Range of the contact rate during reopening stage 2	[9.394, 10.5]
$[\underline{c_{r,3}}, \underline{c_{r,3}}]$	Range of the contact rate during reopening stage 3	[10.5, 11.58]
q_r	Quarantine fraction after reopening	0.3949
$\delta_{I,r}$	Transition rate of symptomatic infected individuals to the quarantined infected class after reopening	0.1378
β^{NV}_{r}	Probability of transmission per contact (non-VOC)	0.1073
β^{V}_{r}	Probability of transmission per contact (VOC)	0.1502

We consider the acceptable de-escalation strategies which meet the constraints

$$D_{ICU}(t) \leq \underline{D_{ICU}} \quad for \quad t_0 < t < t_0 + T,$$

where, $t_0 + T$ is the time corresponding to the end of cost-evaluation and $\underline{D_{ICU}}$ is a capacity of the ICU beds available for COVID-19 patients. We assume that only a portion of ICU beds and hospital wards are available for COVID-19 patients.

A reopening strategy refers to the vectors $(\epsilon_0, \epsilon_1, \epsilon_2)$ determined by $\epsilon_i = t_{i+1} - t_i$, for $i = 1,2,3$. Here, ϵ_0 represents the length of de-escalation phase 0 starting from t_0, 1, 2 and 3 are the duration of the de-escalation phases 1, 2 and 3, respectively. The initiation time of reopening stage 3 is at the time $t_3 = t_2 + \epsilon_2$. Note that ϵ_3 is determined by the equation $\epsilon_0 + \epsilon_1 + \epsilon_2 + \epsilon_3 = T$ and we denote a reopening strategy corresponding to the scenario $(c^j_{r,1}, c^j_{r,2}, c^j_{r,3})$ to be $(\epsilon^j_0, \epsilon^j_1, \epsilon^j_2)$.

In the first simulation, we consider the situation that reopening stages are separated by at least two weeks, so that the minimal 2 week stage switching time is assumed $\epsilon_i \geq 14$, for $i = 0,1,2$. The days of cost-evaluation are set so that the end of the evaluation time corresponds to May 31, 2021.

We solve the following scenario-based stochastic programming model [15] to minimize the intensity of reduced contacts (cost) during de-escalation phases 0, 1, 2 and 3,

$$\sum_j w^j \left(\sum_{i=0}^{3} (\underline{c_{r,3}} - c_{r,i}^j)\epsilon_i^j + ug(\epsilon_0^j, \epsilon_1^j, \epsilon_2^j)\right), \qquad (3)$$

where

$$g(\epsilon_0^j, \epsilon_1^j, \epsilon_2^j) = -log\left(\underline{D_{ICU}} - \max_{t_0<t<t_0+T} D_{ICU}(t)\right),$$

u is set to 10^{-6}, w^j are the likelihood of scenarios. All constraints mentioned above, especially the dynamic transmission model, are included in the above scenario-based stochastic programming model. Furthermore, all decision variables are subject to the non-anticaptivity constraints so as to generate a recourse solution, in which ϵ_0 is made before any uncertainty is revealed, ϵ_1^j are made after $c_{r,1}^j$ is observed and is adapted to it, and ϵ_2^j are made after both $c_{r,1}^j$ and $c_{r,2}^j$ are observed and are adaptive as well. Hence ϵ_0 part of the resource solution is immediately implementable by the policymaker, while ϵ_1^j and ϵ_2^j are path-dependent, providing solutions to all possible future cases present in the model. The solution suite covers favorable cases, as well as unfavorable cases. The decision model incorporates uncertainty and models the adaptive decision process.

The scenario-based model is then implemented in a rolling horizon scheme, i.e., we solve the model and implement the optimal ϵ_0 right away, and near the end of ϵ_0, we solve the model with new information collected during ϵ_0 such as the actual system status and updated distribution of involved random variables, etc. The updated model will yield a new ϵ_0 to be implemented at that time. Though the ϵ_1^j and and ϵ_2^j are never implemented, their existence shows that the ϵ_0 being implemented will not lead to future infeasibility for any scenario considered in the model.

The subsequent sections outline the changes in parameters of the transmission model and stochastic optimization simulation setup according to the simulations where the optimal strategy is adopted on March 14th, May 16th and June 14th, 2021.

Pathways towards mass vaccination should the optimal strategy be initiated on March 14th, 2021

**Supplementary Table 4:** Parameters for COVID-19 transmission dynamics	**Definition**	**Value**

post-reopening in Ontario, Canada (March 14th, 2021). Note*: from data fitting of ICU and ward between December 23, 2020 to March 09, 2021. The initial value for VOC is 2% of E, I, A, S_q, E_q, D_{mild}, $D_{ICU} D_{ward}$ in Supplementary Table 3 and $\epsilon_i \geq 7$, for $i = 0,1,2$.**Parameter**		
c^1	Contact rate between January 9 and February 1	4.6
c^2	Contact rate between February 1 and February 14	5.4
c^3	Contact rate between February 14 and March 14	5.77
$c_{r,0}$	Contact rate during de-escalation phase 0 (since March 14)	5.77
$[\underline{c_{r,1}}, \underline{c_{r,1}}]$	Range of the contact rate during de-escalation phase 1	[6, 7]
$[\underline{c_{r,2}}, \underline{c_{r,2}}]$	Range of the contact rate during de-escalation phase 2	[6.5, 7.5]
$[\underline{c_{r,3}}, \underline{c_{r,3}}]$	Range of the contact rate during de-escalation phase 3	[7, 8]
${\beta^V}_r$	Probability of transmission per contact (VOC)	0.161
h^*	Proportion of hospitalized cases among newly confirmed cases	0.0342
w^*	Proportion of cases admitted to non-ICU units among newly hospitalized cases	0.8731
${b_{ward}}^*$	Rate of transfer from non-ICU units to ICU units	0.0184

Pathways towards mass vaccination should the optimal strategy be initiated on May

16th, 2021

In this set of scenarios, the vaccine rollout parameters were updated to reflect the vaccination program in Ontario (Supplementary Table 5). In the simulations, the contact rates for the de-escalations phases are shown in Supplementary Table 6. The contact rates in each de-escalation phase gradually increase until de-escalation phase 3 (the final phase), which has range [9.5, 10]. This final range was chosen, as these contact rates are representative of contact rates or activity levels close to the estimated pre-pandemic activity levels [4]. The parameters associated with the diagnosed case compartments were also updated in this set of simulations (Supplementary Table 7). In Supplementary Table 7, the parameters associated with model equations (2) are provided for both the resident and the VOC. Given that the ICU capacity for COVID-19 patients was exceeded in early April 2021, we no longer required strategies to meet the constraint

$$D_{ICU}(t) \leq D_{ICU} \text{ for } t_0 < t < t_0 + T.$$

The following stochastic programming model was solved to minimize the intensity of reduced contacts during the de-escalation phases 0, 1, 2 and 3,

$$\sum_j w^j \left(\sum_{i=0}^{3}\left(\overline{c_{r,3}} - c_{r,i}^j\right)\epsilon_i^j + \mu g\left(\epsilon_0^j, \epsilon_1^j, \epsilon_2^j\right)\right) \quad (4)$$

where

$$g(\epsilon_0^j, \epsilon_1^j, \epsilon_2^j) = -\left(\underline{D_{ICU}} - \max_{t_0<t<t_0+T} D_{ICU}(t)\right)^{\kappa},$$

u is set to 10^{-4}, w^j are the likelihood of scenarios and κ equals 3.

Supplementary Table 5: Vaccine rollout parameters reflecting the vaccination program in Ontario. Note*: reach the threshold of 80% of adult's population. $V^{**} = 100{,}000 \times \frac{S}{N}$ per day.

Name	Time Interval	Value (Doses)
v_1	2020-12-23 to 2021-02-14	10,000 per day
v_2	2021-02-14 to 2021-03-13	40,000 per day
v_3	2021-03-13 to 2021-04-04	52,000 per day
v_4	2021-04-05 to 2021-06-06*	100,000 per day
v_5	After 2021-06-06	*V***

Supplementary Table 6: Contact rates prior to the optimal process and the de-escalation phases considered in the optimization. exponential decay* = (8.5-4.5)*exp(-0.297*t)+4.5, t=0 at 12-Apr-2021.

Name	Meaning	Time Interval	Value
C_w	Winter Break	2020-12-23 to 2021-01-09	8.4644
C_s	Stay Home	2021-01-10 to 2021-02-14	4.6993
C_r	Response Framework	2021-02-15 to 2021-03-13	6.5
C_E	Easter	2021-03-14 to 2021-04-11	8.5
C_l	Lockdown	2021-04-12 to 2021-05-16	exponential decay*
C_{r_0}	Phase 0 reopening	Start from 2021-05-03 with	7.77
C_{r_1}	Phase 1 reopening	ϵ_0 days	[8,9]
C_{r_2}	Phase 2 reopening	ϵ_1 days	[8.5,9.5]
C_{r_3}	Phase 3 reopening	$\epsilon_2$days	[9.5,10]

Supplementary Table 7: Estimated model parameters associated with the diagnosed case compartmentalization. In the transmission dynamics model, the hospitalization rate h is a function, defined as $h = h^*(1 - 0.3 \times \frac{V}{S})$, where V is the cumulative number of individuals have been administered vaccine (do not time effective rate 0.7), and h^* is the parameter value in the below table.

Parameter	Definition	Value
**Model parameters before 2021-02-19**		
h^{w*}	Proportion of hospitalized cases	0.0521
h^{VOC*}	Proportion of hospitalized cases (VOC)	0.0126
γ_{ICU}^{w}	Recovery rate of individuals in ICU units (wild)	0.1431
γ_{ICU}^{VOC}	Recovery rate of individuals in ICU units (VOC)	0.0876
w	Proportion of cases admitted to non-ICU	0.9563
b_{ward}^{w}	Rate of transfer from non-ICU units to ICU units (wild)	0.0499
b_{ward}^{VOC}	Rate of transfer from non-ICU units to ICU units (VOC)	0.0641
**Model parameters after 2021-02-19**		
h^{w*}	Proportion of hospitalized cases	0.0655
h^{VOC*}	Proportion of hospitalized cases (VOC)	0.0470
γ_{ICU}^{w}	Recovery rate of individuals in ICU units (wild)	0.0933
γ_{ICU}^{VOC}	Recovery rate of individuals in ICU units (VOC)	0.0853
w	Proportion of cases admitted to non-ICU	0.7872

b_{ward}^{w}	Rate of transfer from non-ICU units to ICU units (wild)	0.0482
b_{ward}^{VOC}	Rate of transfer from non-ICU units to ICU units (VOC)	0.0337

Pathways towards mass vaccination should the optimal strategy be initiated on June 14th, 2021

To consider the impact of vaccination, we modified the transmission dynamics model as follows:

$$\begin{aligned} S' &= -\big(\beta c + cq(1-\beta)\big)\frac{S(I+\theta A)}{N} + \lambda S_q - \chi, \\ E' &= \frac{\beta c(1-q)(S+V_1)(I+\theta A)}{N} - \sigma E, \\ I' &= \sigma\rho E - (\delta_I + \alpha + \gamma_I)I, \\ A' &= \sigma(1-\rho)E - \gamma_A A, \\ S_q{}' &= \frac{(1-\beta)cqS(I+\theta A)}{N} - \lambda S_q, \\ E_q{}' &= \frac{\beta cq(S+V_1)(I+\theta A)}{N} - \delta_q E_q, \\ D' &= \delta_I I + \delta_q E_q - (\alpha + \gamma_D)D, \\ R' &= \gamma_I I + \gamma_A A + \gamma_D D, \\ V_1' &= \chi - \frac{\beta c V_1(I+\theta A)}{N} - lV_1, \\ V_2' &= lV_1. \end{aligned} \tag{5}$$

Note that in the above model, we used the vaccinated population for the "effectively vaccinated populations" in the sense that effectively vaccinated individuals have 100% protection against transmission. The parameter χ, therefore is in fact the daily vaccination rate times the vaccine protection against the transmission. So, if the daily vaccination rate is 60,000 and the vaccine efficacy against transmission is 80%, then χ is 48,000. This assumption can be replaced by more explicitly incorporating "leaky" or "all-or-nothing" vaccines in future studies [10].

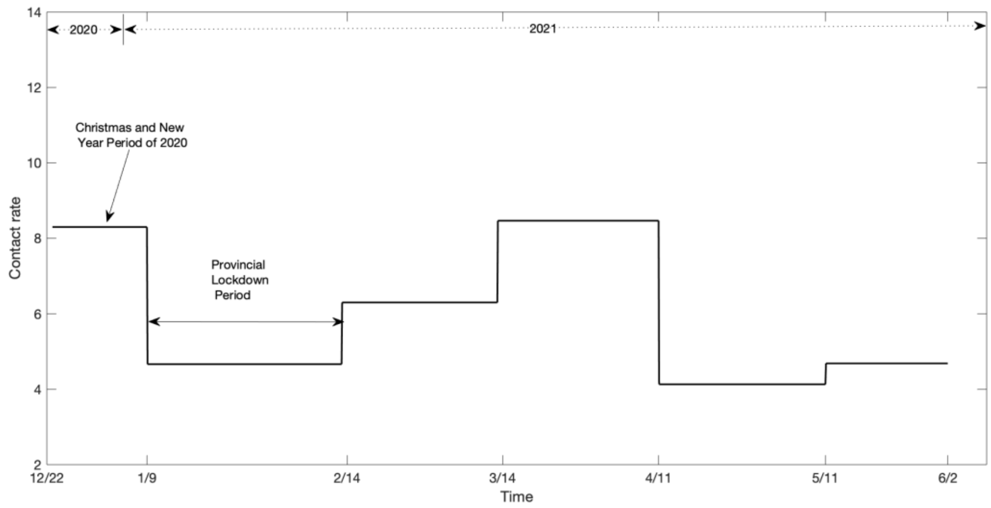

Supplementary Figure 2. Re-estimated activity levels in Ontario during different phases of physical distancing measures from December 22, 2020 till June 2, 2021, using model (5) incorporating vaccination rollout.

Supplementary Table 8: Vaccine rollout parameters reflecting the vaccination program in Ontario. Note*: reach the threshold of 80% of adult's population. $V^{**} = 150{,}000 \times S/N$ per day.

Name	Time Interval	Value (Doses)
v_1	2020-12-23 to 2021-05-26	Data
v_2	2021-05-26 to 2021-06-15	150,000 per day
v_3	2021-06-15 to end	V**

Supplementary Table 9: Contact rates prior to the optimal process and the de-escalation phases considered in the optimization.

Name	Meaning	Time Interval	Value
C_w	Winter Break	2020-12-23 to 2021-01-09	8.3
C_s	Stay Home	2021-01-10 to 2021-02-14	4.6642
C_r	Response Framework	2021-02-15 to 2021-03-13	6.2998
C_E	Easter	2021-03-14 to 2021-04-11	8.4685
C_{L1}	**Lockdown**	2021-04-12 to 2021-05-11	**4.13**
C_{L2}	**Lockdown**	2021-05-12 to 2021-06-13	**4.68**
C_{r_0}	Phase 0 reopening	Start from 2021-06-14 with ϵ_0 days	4.6842
C_{r_1}	Phase 1 reopening	ϵ_1 days	[6,8]
C_{r_2}	Phase 2 reopening	ϵ_2 days	[8,9.5]
C_{r_3}	Phase 3 reopening	ϵ_3 days	[9,11]

Supplementary Table 10: Estimated model parameters associated with the diagnosed case compartmentalization. In the transmission dynamics model, the hospitalization rate h is a function, defined as $h = h^*(1 - 0.3 \times \frac{V}{S})$, where V is the cumulative number of individuals have been administered vaccine (do not time effective rate 0.7), and h^* is the parameter value in the below table.

Model parameters before 2021-02-27		
h^{w*}	Proportion of hospitalized cases	0.0492
h^{VOC*}	Proportion of hospitalized cases (VOC)	0.0140
γ_{ICU}^{w}	Recovery rate of individuals in ICU units (wild)	0.1029

γ_{ICU}^{VOC}	Recovery rate of individuals in ICU units (VOC)	0.0826
w	Proportion of cases admitted to non-ICU	0.9748
b_{ward}^{w}	Rate of transfer from non-ICU units to ICU units (wild)	0.0388
b_{ward}^{VOC}	Rate of transfer from non-ICU units to ICU units (VOC)	0.0527
**Model parameters before 2021-04-11**		
h^{w*}	Proportion of hospitalized cases	0.0647
h^{VOC*}	Proportion of hospitalized cases (VOC)	0.0476
γ_{ICU}^{w}	Recovery rate of individuals in ICU units (wild)	0.0829
γ_{ICU}^{VOC}	Recovery rate of individuals in ICU units (VOC)	0.0903
w	Proportion of cases admitted to non-ICU	0.8343
b_{ward}^{w}	Rate of transfer from non-ICU units to ICU units (wild)	0.05
b_{ward}^{VOC}	Rate of transfer from non-ICU units to ICU units (VOC)	0.0255
**Model parameters after 2021-04-11**		
h^{w*}	Proportion of hospitalized cases	0.0492
h^{VOC*}	Proportion of hospitalized cases (VOC)	0.0394
γ_{ICU}^{w}	Recovery rate of individuals in ICU units (wild)	0.0769
γ_{ICU}^{VOC}	Recovery rate of individuals in ICU units (VOC)	0.0521
w	Proportion of cases admitted to non-ICU	0.9132
b_{ward}^{w}	Rate of transfer from non-ICU units to ICU units (wild)	0.0362
b_{ward}^{VOC}	Rate of transfer from non-ICU units to ICU units (VOC)	0.0552

2 Supplementary results

Pathways should the optimal strategy be implemented on Feb. 14th, 2021

Supplementary Table 11: Optimal de-escalation strategies considering various scenarios for de-escalation phase 0, 1 and 2. Each of the ϵ_1, ϵ_2 and ϵ_3 represents the duration (in days) of de-escalation phase 0, 1 and 2, respectively. Calendar dates presented in the rows of t_1, t_2, t_3 correspond to the date of initiating de-escalation phase 1, 2 and 3.

	s_1	s_2	s_3	s_4	s_5	s_6	s_7	s_8
$c_{1,r}$	7.55	8.12	7.55	8.12	7.55	8.12	7.55	8.12
$c_{2,r}$	9.90	9.90	10.09	10.09	9.90	9.90	10.09	10.09
$c_{3,r}$	10.99	10.99	10.99	10.99	11.18	11.18	11.18	11.18
ϵ_0	55.28	55.28	55.28	55.28	55.28	55.28	55.28	55.28
ϵ_1	14.04	15.93	14.04	15.93	14.04	15.93	14.04	15.93
ϵ_2	24.52	24.56	26.48	25.78	24.52	24.56	26.48	25.78
t_1	10-Apr-2021	10-Apr-2021	10-Apr-2021	10-Apr-2021	10-Apr-2021	10-Apr-2021	10-Apr-2021	10-Apr-2021
t_2	24-Apr-2021	26-Apr-2021	24-Apr-2021	26-Apr-2021	24-Apr-2021	26-Apr-2021	24-Apr-2021	26-Apr-2021
t_3	19-May-2021	21-May-2021	21-May-2021	22-May-2021	19-May-2021	21-May-2021	21-May-2021	22-May-2021

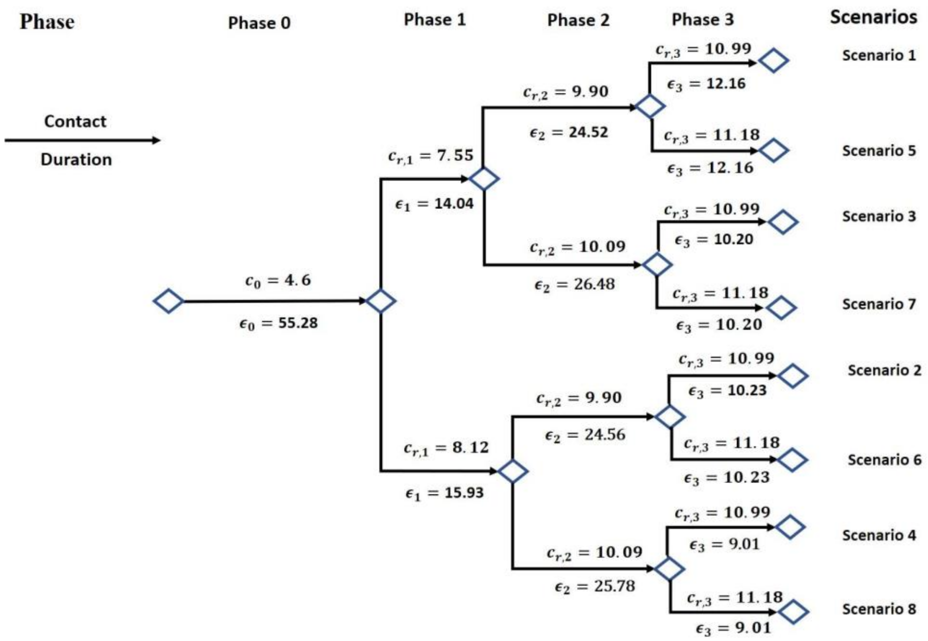

Supplementary Figure 3: A scenario tree with 8 scenarios presented in Supplementary Table 11.

Supplementary Figure 4 shows the histograms of ϵ_0 generated from 100 times simulations. We observe that the resulting ϵ_0 has values of 56 days within $\pm$ 4 days of differences.

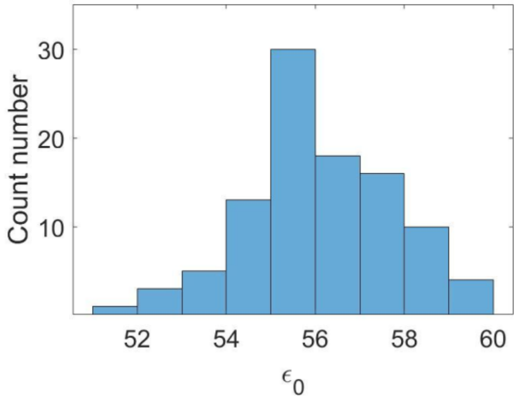

Supplementary Figure 4: Histograms of ϵ_0, the duration of de-escalation phase 0, resulting from 100 simulations. Mean value of ϵ_0 with 8 scenarios is 56.11.

Pathways towards mass vaccination should the optimal strategy be initiated on March 14th, 2021

Supplementary Table 12: Optimal de-escalation strategies considering various scenarios for reopening stage 0, 1 and 2. Each of the ϵ_1, ϵ_2 and ϵ_3 represents the duration (in days)

of de-escalation phase 0, 1 and 2, respectively. Calendar dates presented in the rows of t_1, t_2, t_3 correspond to the date of initiating reopening stage 1, 2 and 3.

	s_1	s_2	s_3	s_4	s_5	s_6	s_7	s_8
$c_{1,r}$	6.46	6.63	6.46	6.63	6.46	6.63	6.46	6.63
$c_{2,r}$	6.96	6.96	7.13	7.13	6.96	6.96	7.13	7.13
$c_{3,r}$	7.73	7.73	7.73	7.73	7.81	7.81	7.81	7.81
ϵ_0	24.47	24.47	24.47	24.47	24.47	24.47	24.47	24.47
ϵ_1	7.64	9.11	7.64	9.11	7.64	9.11	7.64	9.11
ϵ_2	13.28	12.35	13.77	12.73	13.28	12.35	13.77	12.73
t_1	07-Apr-2021	07-Apr-2021	07-Apr-2021	07-Apr-2021	07-Apr-2021	07-Apr-2021	07-Apr-2021	07-Apr-2021
t_2	15-Apr-2021	17-Apr-2021	15-Apr-2021	17-Apr-2021	15-Apr-2021	17-Apr-2021	15-Apr-2021	17-Apr-2021
t_3	28-Apr-2021	29-Apr-2021	29-Apr-2021	29-Apr-2021	28-Apr-2021	29-Apr-2021	29-Apr-2021	29-Apr-2021

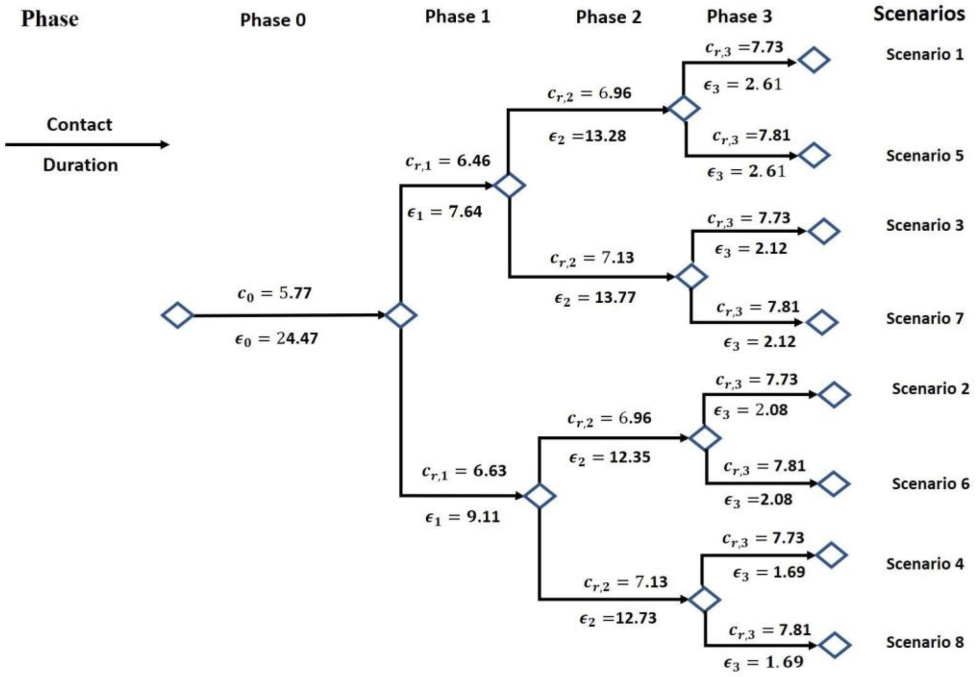

Supplementary Figure 5: A scenario tree with 8 scenarios presented in Supplementary Table 12.

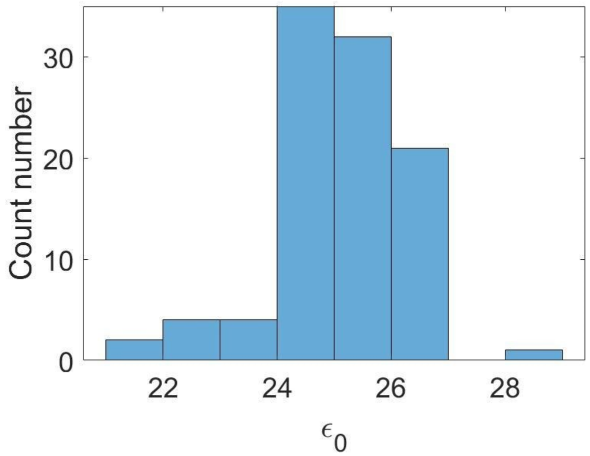

Supplementary Figure 6: Histograms of ϵ_0, the duration of de-escalation phase 0, resulting from 100 simulations. Mean value of 0 with 8 scenarios is 25.02.

Pathways should the optimal strategy be implemented on May 16th, 2021

Supplementary Table 13: Optimal de-escalation strategies considering various scenarios s_1 – s_8 for de-escalation phase 0, 1 and 2. Each of the ϵ_0, ϵ_1 and ϵ_2 represents the duration (in days) of de-escalation phase 0, 1 and 2, respectively. Calendar dates presented in the rows of t_1, t_2, t_3 correspond to the date of initiating de-escalation phase 1, 2 and 3.

	s_1	s_2	s_3	s_4	s_5	s_6	s_7	s_8
$c_{1,r}$	8.46	8.63	8.46	8.63	8.46	8.63	8.46	8.63
$c_{2,r}$	8.96	8.96	9.13	9.13	8.96	8.96	9.13	9.13
$c_{3,r}$	9.73	9.73	9.73	9.73	9.81	9.81	9.81	9.81
ϵ_0	7.0	7.0	7.0	7.0	7.0	7.0	7.0	7.0
ϵ_1	7.0	7.0	7.0	7.0	7.0	7.0	7.0	7.0
ϵ_2	7.0	7.0	7.0	7.0	7.0	7.0	7.0	7.0
t_1	24-May-2021	24-May-2021	24-May-2021	24-May-2021	24-May-2021	24-May-2021	24-May-2021	24-May-2021
t_2	31-May-2021	31-May-2021	31-May-2021	31-May-2021	31-May-2021	31-May-2021	31-May-2021	31-May-2021
t_3	07-Jun-2021	07-Jun-2021	07-Jun-2021	07-Jun-2021	07-Jun-2021	07-Jun-2021	07-Jun-2021	07-Jun-2021

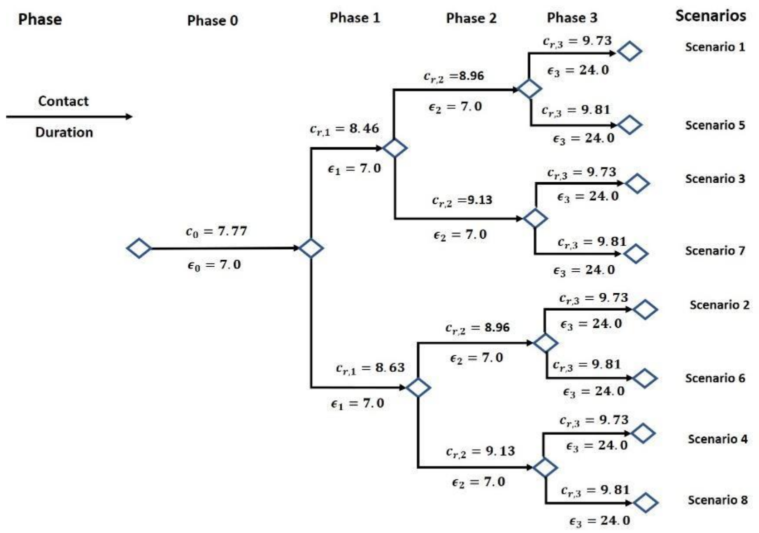

Supplementary Figure 7: Scenario tree with scenarios s_1 – s_8 presented in Supplementary Table 13.

Pathways should the optimal strategy be implemented on June 14th, 2021

In the simulation, the vaccine rollout parameters were updated (Supplementary Table 8), scenarios for the contact rates in each de-escalation phase (Supplementary Table 9) and the model parameters (Supplementary Table 10). As the same in the May 16th, 2021 simulation, the model (5) was solved and the constraint that projected number of ICU beds be below 500 during cost-evaluation was relaxed. The pathways are same for the two scenarios with or without B.1.617 emergence considered.

Supplementary Table 14: Optimal de-escalation strategies considering various scenarios s_1 – s_8 for de-escalation phase 0, 1 and 2. Each of the ϵ_0, ϵ_1 and ϵ_2 represents the duration (in days) of de-escalation phase 0, 1 and 2, respectively. Calendar dates presented in the rows of t_1, t_2, t_3 correspond to the date of initiating de-escalation phase 1, 2 and 3.

	s_1	s_2	s_3	s_4	s_5	s_6	s_7	s_8
$c_{1,r}$	6.23	6.93	6.23	6.93	6.23	6.93	6.23	6.93
$c_{2,r}$	8.17	8.17	8.70	8.70	8.17	8.17	8.70	8.70
$c_{3,r}$	9.23	9.23	9.23	9.23	9.93	9.93	9.93	9.93
ϵ_0	7.0	7.0	7.0	7.0	7.0	7.0	7.0	7.0
ϵ_1	7.0	7.0	7.0	7.0	7.0	7.0	7.0	7.0
ϵ_2	7.0	7.0	7.0	7.0	7.0	7.0	7.0	7.0
t_1	10-Jun-2021	10-Jun-2021	10-Jun-2021	10-Jun-2021	10-Jun-2021	10-Jun-2021	10-Jun-2021	10-Jun-2021
t_2	17-Jun-2021	17-Jun-2021	17-Jun-2021	17-Jun-2021	17-Jun-2021	17-Jun-2021	17-Jun-2021	17-Jun-2021
t_3	24-Jun-2021	24-Jun-2021	24-Jun-2021	24-Jun-2021	24-Jun-2021	24-Jun-2021	24-Jun-2021	24-Jun-2021

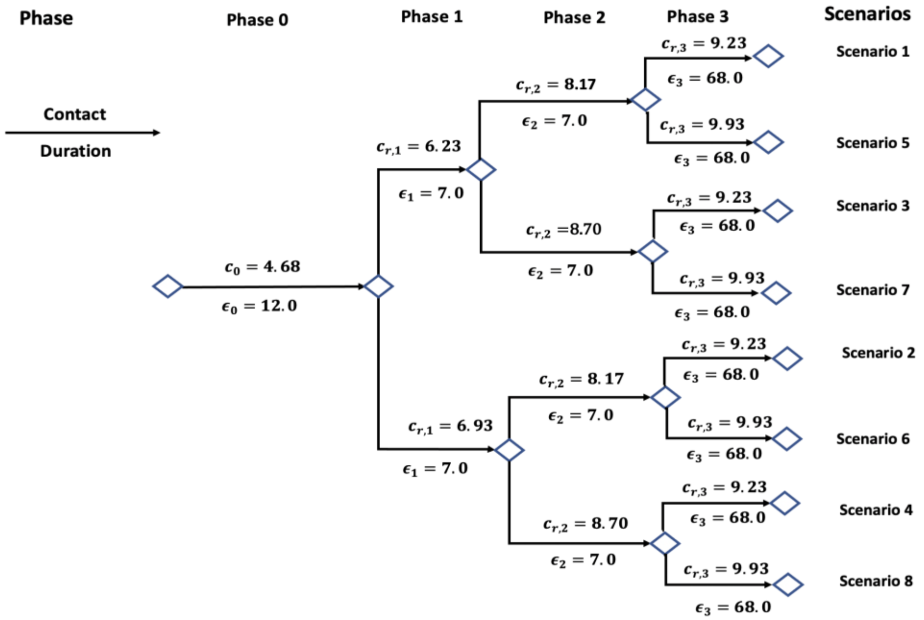

Supplementary Figure 8: Scenario tree with scenarios s_1 – s_8 presented in Supplementary Table 14.
